# A phase Ib study of adavosertib, a selective Wee1 inhibitor, in patients with locally advanced or metastatic solid tumors

**DOI:** 10.1007/s10637-023-01371-6

**Published:** 2023-05-12

**Authors:** Gerald S Falchook, Jasgit Sachdev, Esteban Rodrigo Imedio, Sanjeev Kumar, Ganesh M Mugundu, Suzanne Jenkins, Juliann Chmielecki, Suzanne Jones, David R Spigel, Melissa Johnson

**Affiliations:** 1 Sarah Cannon Research Institute at HealthONE, Denver, CO USA; 2 HonorHealth Research Institute, Scottsdale, AZ USA; 3 Oncology R&D, AstraZeneca, Cambridge, UK; 4 Clinical Pharmacology and Quantitative Pharmacology, CPSS, AstraZeneca, Boston, MA USA; 5 Translational Medicine, Early Research and Development, AstraZeneca, Boston, MA USA; 6 Sarah Cannon Research Institute, Nashville, TN USA; 7 Tennessee Oncology, Nashville, TN USA

**Keywords:** Adavosertib, AZD1775, Cancer, Wee1, Clinical trial, Phase I

## Abstract

Adavosertib selectively inhibits Wee1, which regulates intra-S and G2/M cell-cycle checkpoints. This study investigated dosing schedules for adavosertib monotherapy, determining the maximum tolerated dose (MTD) and recommended Phase II dose (RP2D) in patients with advanced solid tumors.

Patients received oral adavosertib qd or bid on a 5/9 schedule (5 days on treatment, 9 days off) in 14-day cycles, or qd on one of two 5/2 schedules (weekly, or for 2 of 3 weeks) in 21-day cycles. Safety, efficacy, and pharmacokinetic analyses were performed.

Sixty-two patients (female, 64.5%; median age, 61.5 years; most common primary tumors: lung [24.2%], ovary [21.0%]) received treatment (qd schedules, n = 50; bid schedules, n = 12) for 1.8 months (median). Median time to maximum adavosertib concentration was 2.2–4.1 h; mean half-life was 5–12 h. Adverse events (AEs) caused dose reductions, interruptions and discontinuations in 17 (27.4%), 25 (40.3%) and 4 (6.5%) patients, respectively. Most common grade ≥ 3 AEs were anemia, neutropenia (each n = 9, 14.5%) and diarrhea (n = 8, 12.9%). Seven (11.3%) patients experienced 10 treatment-related serious AEs (pneumonia n = 2 [3.2%], dehydration n = 2 [3.2%], anemia n = 1 [1.6%], febrile neutropenia n = 1 [1.6%], and thrombocytopenia n = 1 [1.6%]). Overall objective response rate was 3.4% (2/58); disease control rate was 48.4% (30/62); median progression-free survival was 2.7 months.

MTDs were 125 mg (bid 5/9) and 300 mg (qd 5/9 and 5/2 for 2 of 3 weeks); RP2D was 300 mg (qd 5/2 for 2 of 3 weeks). The safety profile was manageable, acceptable, and generally concordant with the known safety profile.

**Supplementary Information:**

The online version contains supplementary material available at 10.1007/s10637-023-01371-6.

## Introduction

Adavosertib (AZD1775) is a first-in-class selective small-molecule inhibitor of the tyrosine kinase Wee1, a regulator of the intra-S and G2/M cell-cycle checkpoints. Wee1 regulates mitosis and DNA replication via phosphorylation and inhibition of cyclin-dependent kinases (CDK) 1 and 2 [1, 2].

In preclinical studies, Wee1 inhibition caused dysregulation of CDK1 and CDK2, stimulating replication stress and DNA damage, which led to replication catastrophe, DNA damage, and ultimately cell death in various cancer cell lines [3–6]. Adavosertib has been evaluated pre-clinically as a single-agent anticancer therapy in multiple cancer cell lines [7, 8]. Furthermore, in a Phase I study, single-agent activity was shown with adavosertib in patients with advanced solid tumors with BRCA mutations [9], while single-agent activity was also shown with adavosertib in a Phase II study in patients with recurrent uterine serous carcinoma (USC) [10].

Adavosertib may also enhance the cytotoxic effect of chemotherapeutic agents by suppressing the DNA damage response, causing DNA damage to accumulate [11]. The efficacy and safety of various treatment combinations of anticancer agents with adavosertib has been investigated in early-phase clinical trials [12–14].

A previous Phase I adavosertib monotherapy study reported an acceptable and manageable safety and tolerability profile in 80 patients with advanced solid tumors [15]; at an adavosertib dose of 175 mg twice daily (bid; 3 days on followed by 4 days off treatment for 2 of 3 weeks), diarrhea, nausea and fatigue were the most commonly reported AEs (in > 40% of patients) and the most common grade ≥ 3 AEs (in > 5% of patients) [15]. AEs led to treatment discontinuation in 16.3% of patients, dose interruptions in 22.5% of patients, and dose reductions in 11.3% of patients. A further Phase I dose-escalation study demonstrated the safety, tolerability, and preliminary clinical activity of once-daily adavosertib (200, 225, 250, 300, or 400 mg once daily [qd]; 5 days on followed by 2 days off treatment for 2 of 3 weeks) in patients with advanced solid tumors [16].

This study investigated the safety and efficacy of additional treatment schedules to identify the maximum tolerated dose (MTD) and recommended Phase II dose (RP2D) of adavosertib monotherapy in patients with locally advanced or metastatic solid tumors. The effect of a high-fat meal on adavosertib pharmacokinetics was also investigated.

## Methods

### Study design

This was a Phase Ib, multicenter, open-label, dose-finding clinical trial (NCT02610075). The primary objective was to determine the MTD and RP2D of oral adavosertib monotherapy administered qd or bid in patients with locally advanced or metastatic solid tumors. Various doses of adavosertib were assessed: bid 1 (125 mg), bid 2 (150 mg), qd 1 (200 mg), qd 2 (250 mg) and qd 3 (300 mg). Three types of treatment schedules were investigated: cohorts bid 1, bid 2, qd 1.1, qd 2.1 and qd 3.1 received bid or qd treatment on a 5/9 schedule (5 days on treatment, followed by 9 days off) on a 14-day cycle. Cohorts qd 1.2, qd 2.2, and qd 3.2 received treatment on a 5/2 schedule (5 days on followed by 2 days off for 2 of 3 weeks) in a 21-day cycle. Cohorts qd 2.3 and qd 3.3 received treatment on a weekly 5/2 schedule in a 21-day cycle (Table 1). The highest dose level(s) at which less than one-third of evaluable patients (none of 3 patients or 1 of 6 patients) experienced a dose-limiting toxicity (DLT) was declared the MTD.

**Table 1:** Baseline patient demographics and characteristics

Cohort	Adavosertib dose (schedule)	n	Mean age, years (SD)	Female, n (%)	ECOG performance status, n (%)^a^			Number of previous systemic therapy regimens				
					0	1	0		1	2	3	≥ 4
bid 1	125 mg bid (5/9)^b^	6	56.5 (9.2)	3 (50.0)	5 (83.3)	1 (16.7)	1 (16.7)		0	1 (16.7)	0	4 (66.7)
bid 2	150 mg bid (5/9)^b^	6	66.5 (10.9)	4 (66.7)	2 (33.3)	4 (66.7)	1 (16.7)		1 (16.7)	1 (16.7)	1 (16.7)	2 (33.3)
qd 1.1	200 mg qd (5/9)^b^	5	55.2 (11.3)	2 (40.0)	1 (20.0)	4 (80.0)	0		1 (20.0)	0	1 (20.0)	3 (60.0)
qd 1.2	200 mg qd (5/2)^c^	6	58.5 (6.0)	6 (100.0)	1 (16.7)	5 (83.3)	0		0	1 (16.7)	1 (16.7)	4 (66.7)
qd 2.1	250 mg qd (5/9)^b^	4	65.3 (7.6)	2 (50.0)	0	4 (100.0)	0		0	1 (25.0)	2 (50.0)	1 (25.0)
qd 2.2	250 mg qd (5/2)^c^	3	62.0 (6.6)	3 (100.0)	0	3 (100.0)	0		1 (33.3)	0	0	2 (66.7)
qd 2.3	250 mg qd (5/2 weekly)^d^	10	62.1 (12.3)	5 (50.0)	0	10 (100.0)	1 (10.0)		1 (10.0)	1 (10.0)	0	7 (70.0)
qd 3.1	300 mg qd (5/9)^b^	4	51.0 (16.8)	2 (50.0)	0	4 (100.0)	0		0	2 (50.0)	1 (25.0)	1 (25.0)
qd 3.2	300 mg qd (5/2)^c^	16	65.3 (13.3)	12 (75.0)	5 (31.3)	11 (68.8)	0		1 (6.3)	5 (31.3)	2 (12.5)	8 (50.0)
qd 3.3	300 mg (5/2 weekly)^d^	2	51.5 (5.0)	1 (50.0)	0	2 (100.0)	0		0	1 (50.0)	0	1 (50.0)
Total		62	61.0 (11.7)	40 (64.5)	14 (22.6)	48 (77.4)	3 (4.8)		5 (8.1)	13 (21.0)	8 (12.9)	33 (53.2)

^a^ECOG defines ‘0’ as ‘fully active’ and ‘1’ as ‘restricted in physically strenuous activity’; ^b^Cohorts bid 1, bid 2, qd 1.1, 2.1 and 3.1: 5/9 schedule with dosing on days 1–5 in a 14-day cycle; ^c^Cohorts qd 1.2, 2.2, and 3.2: 5/2 schedule with dosing on days 1–5 and 8–12 in a 21-day cycle; ^d^Cohorts qd 2.3 and 3.3: 5/2 weekly schedule with dosing on days 1–5, 8–12 and 15–19 in a 21-day cycle

bid, twice daily; ECOG, Eastern Cooperative Oncology Group; n, number of patients; qd, once daily; SD, standard deviation

The secondary objectives of this study were to evaluate the safety and tolerability, preliminary antitumor activity and pharmacokinetics (PK) of adavosertib monotherapy.

### Patients

Eligible patients were ≥ 18 years of age with an Eastern Cooperative Oncology Group (ECOG) performance status of 0 or 1 and measurable or non-measurable disease (Response Evaluation Criteria in Solid Tumors [RECIST] v1.1) [17]. Histological or cytological evidence of locally advanced or metastatic solid tumors (excluding lymphoma), for which standard therapy did not exist or had proven ineffective or intolerable, was required.

Exclusion criteria included, but were not limited to: use of anticancer drugs within 21 days or 5 half-lives, whichever was shorter, prior to the first adavosertib dose, with a minimum of 10 days between termination of the prior treatment and administration of adavosertib treatment.

Antiemetic prophylaxis was mandatory. Prior to each adavosertib dose, patients received an oral serotonin 5-HT3 antagonist: ondansetron 8 mg bid or granisetron 1 mg bid. For patients receiving the qd dosing schedule, a second dose of antiemetics could be administered 8 h later if nausea and vomiting continued. Oral dexamethasone 4 mg was given on day 1 of each adavosertib dosing period, unless contraindicated or not well tolerated. Aprepitant and fosaprepitant were not permitted because of known drug–drug interactions.

Patients could continue adavosertib treatment until disease progression, unacceptable toxicity, or any other discontinuation criterion was met.

An independent ethics committee/institutional review board approved the final clinical study protocol, including the final version of the informed consent form and any other written information provided to participants. All participants provided written informed consent, and the study was conducted in accordance with the Declaration of Helsinki, the International Council for Harmonisation, good clinical practice, applicable regulatory requirements, and the AstraZeneca policy on bioethics [18].

### Safety

Safety data were recorded throughout the study until 30 days after the last adavosertib treatment, using the National Cancer Institute Common Terminology Criteria for Adverse Events (CTCAE) v4.03.

DLTs were evaluated during the first 28 days (5/9 schedule) or the first 21 days (5/2 schedule) of treatment. Patients who received less than 80% of the planned adavosertib dose for the treatment cycle were not included in the analysis unless it was due to a DLT. DLTs were defined as toxicities related to adavosertib treatment that met at least one of the following criteria: hematologic toxicities, such as neutropenia or thrombocytopenia (grade ≥ 4 for at least 7 days, including infection with febrile neutropenia); neutropenic fever (grade ≥ 3); thrombocytopenia (grade ≥ 3) with bleeding (grade ≥ 2); non-hematologic toxicity (grade ≥ 3); liver function tests – grade ≥ 3 total bilirubin, alanine aminotransferase or aspartate aminotransferase, or alkaline phosphatase lasting for > 48 h, or any change in liver function tests consistent with Hy’s Law; and any other clinically significant and/or unacceptable toxicity that did not respond to supportive care, resulted in a disruption of the dosing schedule of more than 7 days, or was judged to be a DLT by the investigator in collaboration with the medical monitor.

### Efficacy

Preliminary efficacy was assessed using objective response rate (ORR) in patients with measurable disease, disease control rate (DCR) and progression-free survival (PFS), all in accordance with RECIST v1.1 [17]. Responses for ORR required confirmation after at least 4 weeks.

After initiation of treatment, tumor burden was assessed at baseline and every 8 weeks (± 1 week) for the qd 5/9 dosing, and every 9 weeks (± 1 week) for the qd 5/2 treatment schedule, respectively, using computed tomography or magnetic resonance imaging of the chest and abdomen or pelvis.

Archived tumor tissue of patients who consented and for whom a valid test result was obtained (n = 28) was analyzed using the Foundation Medicine (FMI) next-generation sequencing (NGS) platform to assess correlations between genomic profile and clinical outcomes [19].

### Pharmacokinetics

Pharmacokinetic (non-food effect) analyses were performed on blood samples, which were collected pre-dose during treatment cycle 1 on days 1 and 5, and 1, 2, 4, 6, 8, 10 and 24 h after receiving the first adavosertib dose on days 1 and 5. Pre-dose samples were also collected on day 5 of treatment cycle 5.

The area under the plasma concentration–time curve from time 0 to 10 h post-dose (AUC_0–10_) and maximum concentration (C_max_) were determined.

Dose proportionality (non-food effect) of adavosertib AUC_0–10_ and C_max_ for cycle 1, day 1 was assessed across the 125–300 mg dose range using the power model with the linear regression independently incorporating log-transformed AUC_0–10_ and C_max_. The slope estimate (β) and corresponding 90% confidence interval (CI) were calculated, where a β estimate of 1 with 90% CI entirely within the bounds of 0.8–1.25 indicated perfect dose proportionality.

For the preliminary food-effect analysis, blood samples were collected pre-dose and 1, 2, 4, 6, 8 and 10 h after receiving the first adavosertib dose during treatment cycle 1 on day 1 (fasted condition), and during treatment cycle 2 on day 1 (fed condition). Analyses were performed for the bid dosing schedules and RP2D only.

For the fasted condition, patients were required to fast for at least 10 h prior to receiving adavosertib until 4 h post-dose. Patients were allowed glucose and/or juice if they had signs or symptoms of hypoglycemia after receiving adavosertib. Water was restricted from 1 h pre-dose until 1 h post-dose, except for the 240 mL of water administered with treatment.

For the fed condition, patients fasted for at least 10 h prior to receiving adavosertib until 4 h post-dose, with the exception of a high-fat meal eaten in the 30 min before adavosertib administration. If the meal was not completed within 30 min, adavosertib was administered so long as 75% of the meal had been consumed within 45 min of starting the meal. The study was conducted using the Food and Drug Administration standard high-fat meal under fed conditions, which should have a total of 800 to 1000 kcal, with approximately 50% of the caloric content made up from fat [20].

Natural log-transformed AUC and C_max_ were compared between fed/fasted conditions using a mixed effects analysis of variance model, which was fitted separately for each assessed dose. Estimates of the mean difference between treatments and corresponding 90% CI were calculated using a linear mixed effects model with a fixed effect for treatment (fed versus fasted) and a random effect for patients. Back transformed geometric means and 95% CIs were estimated for each condition.

### Statistical methods

Statistical analyses were performed using SAS by Sarah Cannon Development Innovations, LLC under the direction of the AstraZeneca Biometrics Group. Descriptive statistics were used to summarize the safety, PK and preliminary antitumor activity data by treatment cohort.

Patients who received at least one dose of adavosertib were included in the full analysis set, which was used for safety and efficacy evaluations. The per protocol analysis set was defined as a subset of the full analysis set, excluding subjects having an important protocol deviation, to be used for analyses of efficacy endpoints if appropriate. There were no important protocol deviations; therefore, the patient groups included in the safety and efficacy evaluations were the same.

All patients who received at least one adavosertib dose and provided at least one PK sample were included in the PK analysis.

## Results

A total of 65 patients enrolled in the study. Of these, 62 received treatment (mean age [standard deviation (SD)], 61.0 [11.7] years; 40 [64.5%] women; Table 1), with a median treatment duration of 1.77 months. Most patients (48 [77.4%]) had an ECOG performance status of 1, meaning that they were restricted in performing physically strenuous activity. The majority of patients (57 [91.9%]) were Caucasian. Prior to the study, 59 (95.2%) patients had received systemic therapy regimens (overall median, 4; range, 0–15). Forty-two (67.7%) patients had received radiotherapy, and 47 (75.8%) had undergone disease-related surgery.

The most common (≥ 10% of patients) primary diagnoses were lung (24.2%), ovarian (21.0%) and uterine (12.9%) cancer in 15, 13 and 8 patients, respectively.

All patients received antiemetics during the study. Of these, 49 (79.0%) patients received serotonin 5-HT3 antagonists (granisetron, granisetron hydrochloride, ondansetron, ondansetron hydrochloride, palonosetron hydrochloride) and 48 (77.4%) patients received glucocorticoids (dexamethasone, fluticasone propionate, hydrocortisone, methylprednisolone, prednisolone, prednisone).

Seventeen (27.4%) patients took prohibited concomitant medication during study treatment. No important protocol deviations were reported.

### Safety

In total, 60 (96.8%) patients experienced at least one AE. The most commonly occurring all-grade AEs (in ≥ 30% of patients) were diarrhea in 32 (51.6%) patients, nausea and fatigue in 29 (46.8%) patients each, and dehydration in 23 (37.1%) patients. An overview of all-grade AEs occurring in ≥ 10% of patients per dosing schedule is provided in Supplementary Table S1.

Fifty-six (90.3%) patients experienced at least one AE related to adavosertib treatment (Supplementary Table S2). Of these, the three most commonly occurring AEs were diarrhea in 31 (50.0%), nausea in 26 (41.9%), and fatigue in 24 (38.7%) patients. Grade ≥ 3 AEs were experienced by 39 (62.9%) patients, of whom 24 (38.7%) had at least one treatment-related grade ≥ 3 AE (Table 2). The most common grade ≥ 3 AEs (occurring in ≥ 10% of patients) were anemia, neutropenia, and diarrhea in 9 (14.5%), 9 (14.5%), and 8 (12.9%) patients, respectively.

**Table 2:** AEs with severity grade ≥ 3 and DLTs

Treatment group	Adavosertib dose (schedule)	Any grade ≥ 3 AE, n (%)	Any treatment-related grade ≥ 3 AE, n (%)	DLT evaluable patients,^a^ n (%)	Any DLT, n (%)	DLTs:^b^ AE preferred term (CTCAE grade, G)
*bid 1 (n = 6)*	*125 mg bid (5/9)* ^c^	*4 (66.7)*	*1 (16.7)*	*6 (100.0)*	*0*	–
bid 2 (n = 6)	150 mg bid (5/9)^c^	5 (83.3)	3 (50.0)	6 (100.0)	2 (33.3)	Diarrhea (G3); nausea (G2); dehydration (G3)
qd 1.1 (n = 5)	200 mg qd (5/9)^c^	1 (20.0)	1 (20.0)	5 (100.0)	0	–
qd 1.2 (n = 6)	200 mg qd (5/2)^d^	3 (50.0)	2 (33.3)	5 (83.3)	0	–
qd 2.1 (n = 4)	250 mg qd (5/9)^c^	2 (50.0)	0	4 (100.0)	0	–
qd 2.2 (n = 3)	250 mg qd (5/2)^d^	3 (100.0)	3 (100.0)	3 (100.0)	0	–
qd 2.3 (n = 10)	250 mg qd (5/2 weekly)^e^	5 (50.0)	4 (40.0)	9 (90.0)	2 (22.2)	Thrombocytopenia (G3); neutropenia (G3); thrombocytopenia (G2)
*qd 3.1 (n = 4)*	*300 mg qd (5/9)* ^c^	*3 (75.0)*	*1 (25.0)*	*4 (100.0)*	*0*	–
**qd 3.2 (n = 16)**	**300 mg qd (5/2)** ^**d**^	**12 (75.0)**	**8 (50.0)**	**15 (93.8)**	**2 (13.3)**	**Nausea (G2); weight decreased (G1); pneumonia (G3)**
qd 3.3 (n = 2)	300 mg (5/2 weekly)^e^	1 (50.0)	1 (50.0)	2 (100.0)	2 (100.0)	Thrombocytopenia (G4); nausea (G2); vomiting (G2)
Total (N = 62)		39 (62.9)	24 (38.7)	59 (95.2)	8 (13.6)	12

The MTDs are indicated with italics and the RP2D indicated with bold. ^a^Patients who received less than 80% of treatment during cycle 1 (5/2 schedule) or cycles 1 and 2 (28 days for the 5/9 schedule) were not considered evaluable, unless they experienced a DLT confirmed by the scientific research team; ^b^Some patients experienced more than one DLT. Three patients discontinued prior to receiving adavosertib and were not included in the DLT analysis set; ^c^Cohorts bid 1, bid 2, qd 1.1, 2.1 and 3.1: 5/9 schedule with dosing on days 1–5 in a 14-day cycle; ^d^Cohorts qd 1.2, 2.2, and 3.2: 5/2 schedule with dosing on days 1–5 and 8–12 in a 21-day cycle; ^e^Cohorts qd 2.3 and 3.3: 5/2 weekly schedule with dosing on days 1–5, 8–12 and 15–19 in a 21-day cycle. AE, adverse event; CTCAE, Common Terminology Criteria for Adverse Events; DLT, dose-limiting toxicity

Fifty serious AEs (SAEs) were reported in 25 (40.3%) patients. Of these, seven (11.3%) patients had 10 treatment-related SAEs: pneumonia n = 2 [3.2%], dehydration n = 2 [3.2%], anemia n = 1 [1.6%], febrile neutropenia n = 1 [1.6%], and thrombocytopenia n = 1 [1.6%]. The death of one patient in cohort qd 3.3 was attributed by the investigator to both disease progression and an SAE of treatment-unrelated grade 5 sepsis.

Overall, 12 DLTs were reported in 8 (13.3%) patients (Table 2). DLTs were reported in the bid 2, qd 2.3, qd 3.2 and qd 3.3 cohorts. Thrombocytopenia and nausea occurred most frequently (each n = 3); all other DLTs (dehydration, neutropenia, weight decrease, pneumonia, diarrhea, and vomiting) only occurred once. Distinct MTDs were identified in the bid 1 (125 mg [bid 5/9]), qd 3.1 (300 mg [qd 5/9]) and qd 3.2 (300 mg [qd 5/2]) cohorts; the MTD of 300 mg (qd 5/2) (cohort qd 3.2) was selected as the RP2D (Table 2).

AEs led to dose reductions in 17 (27.4%) patients. The most commonly occurring AEs leading to dose reductions (in ≥ 5% of patients) were thrombocytopenia in 5 (8.1%) patients and neutropenia in 4 (6.5%) patients. AEs led to dose interruptions in 25 (40.3%) patients. The most common AEs leading to interruptions in ≥ 5% of patients were diarrhea, fatigue, thrombocytopenia and dehydration, which occurred in 4 (6.5%) patients each. Overall, 4 (6.5%) patients from different cohorts discontinued treatment because of the following AEs: grade 1 alopecia (qd 1.2); grade 2 fatigue and myalgia (qd 2.3); grade 4 thrombocytopenia (qd 3.1); and grade 2 abdominal pain, diarrhea and nausea (qd 3.3).

### Efficacy

**Fig. 1:**
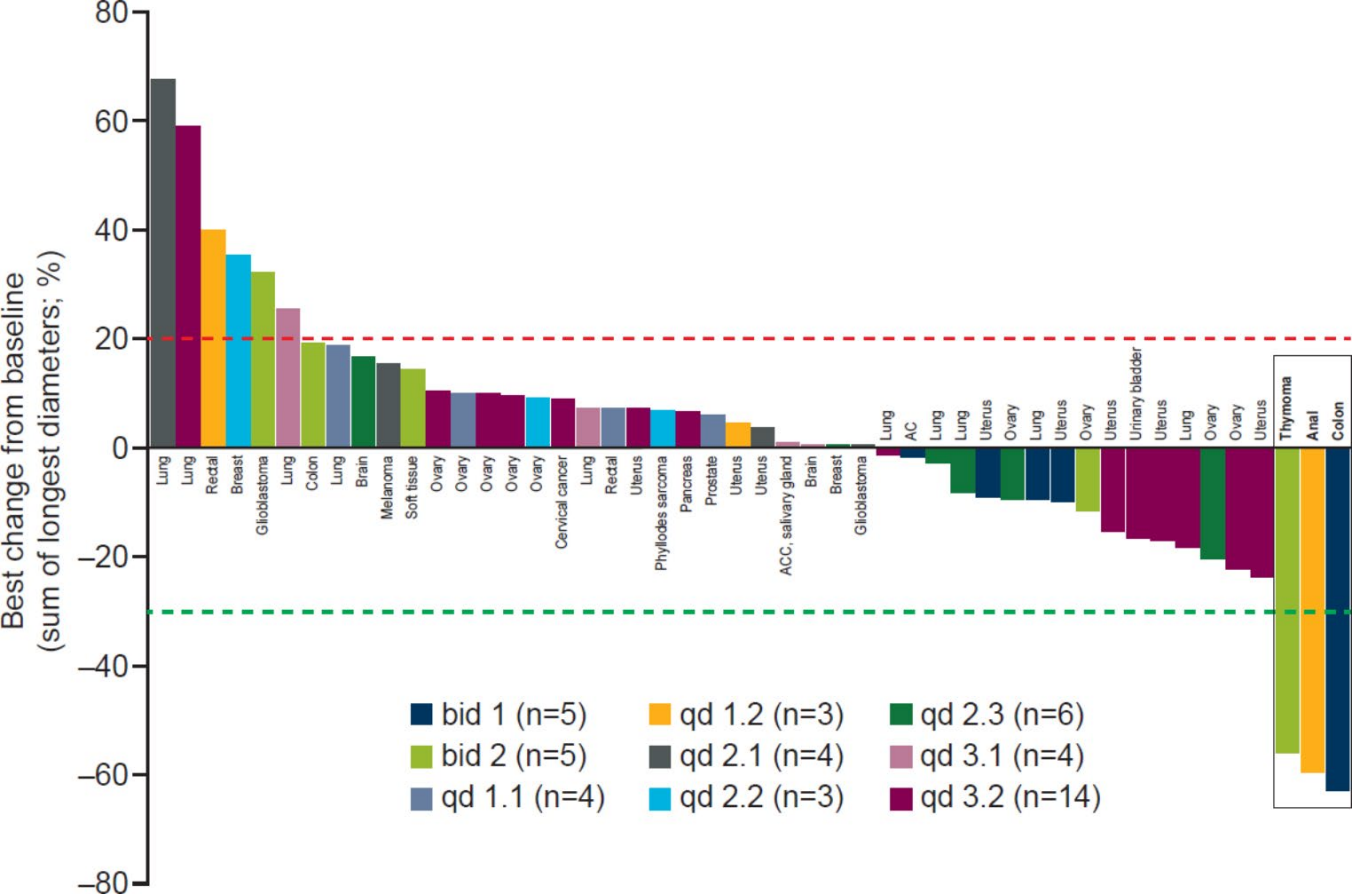
**Best percentage change from baseline in target lesion size across cohorts treated with oral adavosertib** Best change in target lesion size is defined as the maximum reduction from baseline or the minimum increase from baseline in the absence of a reduction (RECIST v1.1). Forty-eight patients, who had measurable disease at baseline and at least one post-baseline scan, were included in this analysis. The three bars on the right depict: one patient with colon cancer (bid 1) who had a partial response in the target lesion but progressive disease in non-target lesions (this patient’s objective response was evaluated as progressive disease); two patients with a confirmed partial response, one had anal cancer (qd 1.2) and the other a thymoma (bid 2) AC, adrenocortical carcinoma; ACC, adenoid cystic carcinoma

Overall ORR was 3.4% (two of the 58 patients with measurable disease at baseline; 95% CI, 0.4–11.9). The DCR was 48.4% (30 of 62 patients; 95% CI, 35.5–61.4). None of the patients had confirmed (or unconfirmed) complete responses. Two patients had a confirmed partial response in the target lesions: 1 patient with thymoma in the bid 2 cohort had a duration of response of 3.7 months, and 1 patient with anal carcinoma in the qd 1.2 cohort had a duration of response of 6.9 months. The best percentage change in target lesion size was assessed for 48 patients (patients with measurable disease at baseline who had at least one post-baseline scan). The two patients with confirmed partial responses in their target lesions and one patient who had a partial response in their target lesion (but due to progressive disease in non-target lesions was evaluated as having an objective response of progressive disease) experienced a best change from baseline of approximately − 60% (Fig. 1).

Stable disease was recorded in 28 (45.2%) patients (maintained for at least 8 weeks for qd 5/2 schedules, or at least 7 weeks for other schedules) and in 21 (33.9%) patients for at least 12 weeks (Table 3). Thirty (48.4%) patients experienced progressive disease. Two (3.2%) patients, in groups qd 1.2 and qd 2.3, were not evaluable because of incomplete data.

**Table 3:** Response to adavosertib treatment

Cohort	Adavosertib dose (schedule)	ORR, CP + PR/n (%)	DCR, CP + PR + SD/n (%)^d^	SD^e^				Median PFS, months (95% CI)
				≥ 7 or 8 weeks^f^	> 12 weeks	< 12 weeks	12 weeks, not confirmed	
bid 1 (n = 6)	125 mg bid (5/9)	0^a^	4 (66.7)	4 (66.7)	3 (50.0)	0	1 (16.7)	3.7 (1.5–5.5)
bid 2 (n = 6)	150 mg bid (5/9)	1/5 (20.0)^b^	2 (33.3)	1 (16.7)	1 (16.7)	0	0	1.9 (1.0–NC)
qd 1.1 (n = 5)	200 mg qd (5/9)	0	2 (40.0)	2 (40.0)	2 (40.0)	0	0	2.0 (0.6–NC)
qd 1.2 (n = 6)	200 mg qd (5/2)	1/5 (20.0)^b^	2 (33.3)	1 (16.7)	1 (16.7)	0	0	2.7 (1.2–9.0)
qd 2.1 (n = 4)	250 mg qd (5/9)	0	1 (25.0)	1 (25.0)	1 (25.0)	0	0	1.9 (1.8–4.1)
qd 2.2 (n = 3)	250 mg qd (5/2)	0	2 (66.7)	2 (66.7)	2 (66.7)	0	0	3.7 (1.2–11.0)
qd 2.3 (n = 10)	250 mg qd (5/2 weekly)	0	3 (30.0)	3 (30.0)	2 (20.0)	0	1 (10.0)	2.1 (0.6–4.1)
qd 3.1 (n = 4)	300 mg qd (5/9)	0	3 (75.0)	3 (75.0)	2 (50.0)	0	1 (25.0)	7.6 (2.7–9.3)
qd 3.2 (n = 16)	300 mg qd (5/2)	0	11 (68.8)	11 (68.8)	7 (43.8)	1 (6.3)	3 (18.8)	3.2 (2.1–5.0)
qd 3.3 (n = 2)	300 mg (5/2 weekly)	0	0	0	0	0	0	2.6 (NC–NC)
Total (N = 62)		2/58^c^ (3.4)	30/62 (48.4)	28/62 (45.2)	21/62 (33.9)	1/62 (1.6)	6/62 (9.7)	2.7 (2.0–3.8)

None of the patients had a complete response. ^a^One patient in the bid 1 cohort had a partial response in the target lesion but progressive disease in non-target lesions; ^b^Patients with a partial response had anal cancer (qd 1.2) and a thymoma (bid 2); ^c^Number of patients with measurable disease at baseline. Four patients in the bid 2, qd 1.2, qd 2.3 and qd 3.2 cohorts did not have measurable target lesions at baseline; ^d^DCR was defined as the proportion of patients with a confirmed (after 4 weeks) BOR of CR or PR; or a BOR of SD for at least 8 weeks for qd 5/2 schedules, or at least 7 weeks for other schedules; ^e^SD > 12 weeks indicates confirmed SD for more than 12 weeks; SD < 12 weeks means that SD was achieved as per protocol definition, but the disease progressed on or before week 12; 12 weeks not confirmed indicates that SD (BOR) was achieved as per protocol definition, but it is unknown if it lasted for 12 weeks or more; ^f^SD for at least 8 weeks for qd 5/2 schedules, or at least 7 weeks for other schedules). BOR, best objective response; CR, complete response; NC, not calculable; PR, partial response; SD, stable disease

Overall median PFS was 2.7 months (95% CI, 2.0–3.8) (Table 3). At the time of data cutoff, 47 (75.8%) patients had experienced an event, 14 (22.6%) patients were alive and progression-free, and 1 (1.6%) patient had been censored due to death. Of 62 patients, 19.8% (95% CI 10.0–32.2) were progression-free at 6 months after receiving their first dose of adavosertib.

At the time of data cutoff (2 years and 8 months after the first patient received adavosertib treatment and 10 months after the last patient received treatment), 14 (22.6%) patients were alive and progression-free.

The NGS profiles correlated to clinical outcome are summarized in Supplementary Fig. S1.

### Pharmacokinetics

Adavosertib was steadily absorbed (median time to C_max_, 2.2–4.1 h) and slowly eliminated (mean half-life, 5–12 h), based on single-dose data. Adavosertib accumulated in plasma after 150 mg bid dosing, with geometric mean accumulation ratios of approximately 2.4. However, adavosertib qd dosing resulted in relatively minimal accumulation with geometric mean accumulation ratios of approximately 1.2 to 1.9 following doses of 200 to 300 mg.

Steady-state concentration at the RP2D (300 mg qd 5/2) was above the half-maximal inhibitory concentration (IC_50_) of phosphorylated CDK1 and within the target range (500–1000 nM) for approximately 10 h post-dose (Supplementary Fig. S2). This was based on preclinical internal data that predicted 300 mg qd as having the greatest cell kill activity and most tumor shrinkage.

Systemic exposure to adavosertib appeared to be more than dose proportional (Supplementary Fig. S3). Power model estimates for cycle 1, day 1 were: AUC_0–10_ β = 1.31 (90% CI, 1.03–1.59) (Supplementary Fig. S3a); and C_max_ β = 1.31 (90% CI, 1.06–1.56) (Supplementary Fig. S3b).

Administration of a single dose of adavosertib with a high-fat meal did not appear to impact the C_max_ or AUC_0–10_ (Table 4). Geometric mean ratios of fed/fasted conditions ranged from 0.911 to 1.089 with all 90% CIs containing unity (Table 4). Based on these findings, there appeared to be no pharmacokinetic impact of administering adavosertib with a high-fat meal.

**Table 4:** Summary of food-effect analyses for the adavosertib bid dosing schedules and RP2D

Cohort	Parameter (units)	n	Fed	Fasted	Fed versus fasted
			Geometric LS mean (95% CI)	Geometric LS mean (95% CI)	Geometric mean ratio (90% CI)
bid 1 125 mg bid (5/9)	AUC_0–10_ (h*nmol/L)	5	1806.4 (1104.0, 2955.9)	1658.8 (1017.1, 2705.3)	1.089 (0.991, 1.197)
	C_max_ (nmol/L)	6	276.2 (204.9, 372.3)	256.7 (190.4, 346.1)	1.076 (0.938, 1.234)
bid 2 150 mg bid (5/9)	AUC_0–10_ (h*nmol/L)	5	3177.3 (1525.3, 6618.2)	3228.0 (1698.9, 6133.3)	0.984 (0.661, 1.467)
	C_max_ (nmol/L)	6	424.2 (273.1, 658.7)	464.3 (303.7, 710.1)	0.913 (0.728, 1.146)
qd 3.2 300 mg qd (5/2)	AUC_0–10_ (h*nmol/L)	3	5664.9 (66.0, 485942.8)	5340.4 (65.0, 439027.6)	1.061 (0.624, 1.802)
	C_max_ (nmol/L)	4	746.6 (261.4, 2132.4)	819.4 (305.5, 2198.0)	0.911 (0.548, 1.514)

AUC_0–10_, area under the concentration–time curve from 0 to 10 h; CI, confidence interval; C_max,_ maximum concentration; LS, least-squares; n, number of patients with evaluable PK data in both fed and fasted states (PK analysis set)

## Discussion

In this Phase Ib, open-label, dose-finding clinical study, we described the MTDs for different schedules, 125 mg (bid 5/9) and 300 mg (qd 5/2 and 5/9), and identified the RP2D of adavosertib monotherapy to be 300 mg (qd 5/2). Steady-state concentration at 300 mg (qd 5/2) was above the IC_50_ of phosphorylated CDK1 and within the target range (500–1000 nM) for approximately 10 h post-dose.

A previous Phase I dose-escalation study with once-daily adavosertib monotherapy in patients with advanced solid tumors identified the RP2D to be 300 mg qd 5/2 for 2 of 3 weeks [16]. This is consistent with the RP2D described in our study, which assessed a wider range of once-daily and twice-daily dosing schedules in patients with advanced solid tumors.

With the mandatory use of antiemetic prophylaxis, we demonstrated a safety profile broadly consistent with earlier monotherapy studies [9, 15, 16]. The safety profile was considered acceptable and manageable in this group of heavily pre-treated patients (median of four prior systemic treatment regimens). For future and ongoing studies, prophylaxis for common AEs and early aggressive management of symptoms are recommended to improve the tolerability of, and adherence to, adavosertib.

Adavosertib monotherapy given in a range of exploratory doses showed minor antitumor activity in this heavily pre-treated group of patients with diverse tumor types. Stable disease for at least 8 weeks for qd 5/2 schedules, or at least 7 weeks for other schedules was observed in 28 (45.2%) patients and in 21 (33.9%) patients for more than 12 weeks; long-term clinical studies should build upon these findings. Evidence of efficacy with adavosertib monotherapy has also been shown in other studies. A Phase II study of adavosertib (300 mg qd on days 1–5 and 8–12 of a 21-day cycle) monotherapy in women with recurrent USC reported an ORR of 29.4% (95% CI 15.1–47.5) with a median PFS of 6.1 months [10]. Additional trials have shown additive effects of combining adavosertib with other anticancer agents in advanced solid tumors. A Phase II randomized controlled trial combining adavosertib (175 mg qd on days 1, 2, 8, 9, 15, and 16 of a 28-day cycle) with gemcitabine in women with platinum-resistant or platinum-refractory recurrent ovarian cancer reached its predefined primary endpoint of increasing PFS (4.6 months; 95% CI 3.6–6.4) vs. the placebo plus gemcitabine arm (3.0 months; 95% CI 1.8–3.8) [21]. An open-label four-arm Phase II study of adavosertib (2 days on/5 days off or 3 days on/4 days off in six cohorts from 175 mg qd to 225 mg bid) with carboplatin in patients with platinum-resistant ovarian cancer showed preliminary efficacy, with a response rate of 66.7% [22]. A randomized Phase II study of adavosertib (300 mg qd on days 1–5 and 8–12 or 150 mg bid on days 1–3 and 8–10 of a 21-day cycle) with or without a poly (ADP-ribose) polymerase (PARP) inhibitor (PARPi) in patients with recurrent ovarian cancer with progressive disease while receiving PARPi showed an ORR of 23% (90% CI 12–38) with adavosertib alone vs. 29% (90% CI 16–44) with combination therapy [23]. A recent Phase Ib trial indicated that alternating treatment with adavosertib and olaparib may be more effective and better tolerated than concurrent drug administration in patients with DNA damage response aberrant advanced tumors [24]. These findings suggest that combining Wee1 inhibition with other agents may be adequate to overcome the resistance to treatment that is observed in certain tumor types.

There was no apparent pharmacokinetic impact of administering adavosertib with a high-fat meal in this assessment. This was confirmed by and is in accordance with findings by Någård et al. [25], who reported similar findings in a randomized, open-label, two-period, two-sequence crossover study.

In conclusion, the RP2D of adavosertib monotherapy was 300 mg (qd 5/2 for 2 of 3 weeks). The safety profile was manageable and consistent with the known safety profile, with limited antitumor activity.

## Electronic supplementary material

Supplementary Material 1

Supplementary Material 2

Supplementary Material 3

Supplementary Material 4

## Data Availability

Data underlying the findings described in this manuscript may be obtained in accordance with AstraZeneca’s data sharing policy described at. https://astrazenecagrouptrials.pharmacm.com/ST/Submission/Disclosure.

## Code Availability

N/A.
