# Supplementary material for: A phase Ib study of adavosertib, a selective Wee1 inhibitor, in patients with locally advanced or metastatic solid tumors
Source: Invest New Drugs. 2023 May 12;41(3):493–502. doi: 10.1007/s10637-023-01371-6 (PMC10289903; doi:10.1007/s10637-023-01371-6)

A Phase Ib study of adavosertib, a selective Wee1 inhibitor, in patients with locally advanced or metastatic solid tumors

*Investigational New Drugs*

Gerald S Falchook,^1^ Jasgit Sachdev,^2^ Esteban Rodrigo Imedio,^3^ Sanjeev Kumar,^3^ Ganesh M Mugundu,^4^ Suzanne Jenkins,^3^ Juliann Chmielecki,^5^ Suzanne Jones,^6^ David R Spigel,^6,7^ Melissa Johnson^6,7^

1Sarah Cannon Research Institute at HealthONE, Denver, CO, USA; 2HonorHealth Research Institute, Scottsdale, AZ, USA; 3Oncology R&D, AstraZeneca, Cambridge, UK; 4Clinical Pharmacology and Quantitative Pharmacology, CPSS, AstraZeneca, Boston, MA, USA; 5Translational Medicine, Early Research and Development, AstraZeneca, Boston, MA, USA; 6Sarah Cannon Research Institute, Nashville, TN, USA; 7Tennessee Oncology, Nashville, TN, USA

**Corresponding author: Gerald Falchook**Email: gerald.falchook@sarahcannon.com

# Supplementary information

**Supplementary Table S1** Most common (≥10% of patients) treatment-emergent adverse events

| **AEs, n (%) MedDRA (v20.1) preferred term** | **Treatment groups** | | | | | | | | | | |
| --- | --- | --- | --- | --- | --- | --- | --- | --- | --- | --- | --- |
|  | **bid 1 (n=6)** | **bid 2 (n=6)** | **qd 1.1 (n=5)** | **qd 1.2 (n=6)** | **qd 2.1  (n=4)** | **qd 2.2  (n=3)** | **qd 2.3  (n=10)** | **qd 3.1  (n=4)** | **qd 3.2  (n=16)** | **qd 3.3  (n=2)** | **Total  (N=62)** |
| Any AE | 6 (100) | 6 (100) | 5 (100) | 6 (100) | 4 (100) | 3 (100) | 8 (80.0) | 4 (100) | 16 (100.0) | 2 (100.0) | 60 (96.8) |
| Diarrhea | 2 (33.3) | 5 (83.3) | 1 (20.0) | 3 (50.0) | 1 (25.0) | 2 (66.7) | 7 (70.0) | 0 | 9 (56.3) | 2 (100.0) | 32 (51.6) |
| Fatigue | 5 (83.3) | 5 (83.3) | 2 (40.0) | 1 (16.7) | 0 | 2 (66.7) | 3 (30.0) | 2 (50.0) | 9 (56.3) | 0 | 29 (46.8) |
| Nausea | 3 (50.0) | 3 (50.0) | 1 (20.0) | 4 (66.7) | 0 | 2 (66.7) | 3 (30.0) | 2 (50.0) | 9 (56.3) | 2 (100.0) | 29 (46.8) |
| Dehydration | 2 (33.3) | 4 (66.7) | 0 | 2 (33.3) | 0 | 2 (66.7) | 5 (50.0) | 1 (25.0) | 7 (43.8) | 0 | 23 (37.1) |
| Anemia | 0 | 1 (16.7) | 0 | 2 (33.3) | 1 (25.0) | 1 (33.3) | 2 (20.0) | 1 (25.0) | 8 (50.0) | 0 | 16 (25.8) |
| Edema peripheral | 3 (50.0) | 1 (16.7) | 0 | 3 (50.0) | 1 (25.0) | 0 | 2 (20.0) | 2 (50.0) | 4 (25.0) | 0 | 16 (25.8) |
| Vomiting | 1 (16.7) | 2 (33.3) | 0 | 3 (50.0) | 0 | 2 (66.7) | 2 (20.0) | 0 | 5 (31.3) | 1 (50.0) | 16 (25.8) |
| Abdominal pain | 2 (33.3) | 1 (16.7) | 1 (20.0) | 2 (33.3) | 1 (25.0) | 0 | 2 (20.0) | 1 (25.0) | 4 (25.0) | 1 (50.0) | 15 (24.2) |
| Constipation | 2 (33.3) | 2 (33.3) | 1 (20.0) | 2 (33.3) | 0 | 1 (33.3) | 2 (20.0) | 1 (25.0) | 4 (25.0) | 0 | 15 (24.2) |
| Decreased appetite | 2 (33.3) | 2 (33.3) | 0 | 1 (16.7) | 0 | 1 (33.3) | 3 (30.0) | 0 | 5 (31.3) | 0 | 14 (22.6) |
| Dizziness | 1 (16.7) | 1 (16.7) | 1 (20.0) | 2 (33.3) | 0 | 0 | 2 (20.0) | 2 (50.0) | 4 (25.0) | 1 (50.0) | 14 (22.6) |
| Back pain | 2 (33.3) | 2 (33.3) | 0 | 1 (16.7) | 0 | 1 (33.3) | 2 (20.0) | 1 (25.0) | 4 (25.0) | 0 | 13 (21.0) |
| Myalgia | 1 (16.7) | 0 | 1 (20.0) | 3 (50.0) | 0 | 0 | 2 (20.0) | 1 (25.0) | 3 (18.8) | 1 (50.0) | 12 (19.4) |
| Hypokalemia | 1 (16.7) | 1 (16.7) | 0 | 0 | 0 | 1 (33.3) | 3 (30.0) | 0 | 4 (25.0) | 1 (50.0) | 11 (17.7) |
| Asthenia | 2 (33.3) | 1 (16.7) | 2 (40.0) | 0 | 0 | 1 (33.3) | 0 | 0 | 4 (25.0) | 0 | 10 (16.1) |
| Neutropenia | 0 | 2 (33.3) | 1 (20.0) | 1 (16.7) | 0 | 0 | 1 (10.0) | 0 | 4 (25.0) | 1 (50.0) | 10 (16.1) |
| Pyrexia | 0 | 2 (33.3) | 1 (20.0) | 1 (16.7) | 1 (25.0) | 2 (66.7) | 0 | 0 | 3 (18.8) | 0 | 10 (16.1) |
| Thrombocytopenia | 1 (16.7) | 0 | 0 | 0 | 0 | 1 (33.3) | 3 (30.0) | 1 (25.0) | 3 (18.8) | 1 (50.0) | 10 (16.1) |
| Cough | 1 (16.7) | 1 (16.7) | 0 | 1 (16.7) | 2 (50.0) | 1 (33.3) | 0 | 1 (25.0) | 2 (12.5) | 0 | 9 (14.5) |
| Dyspnea | 1 (16.7) | 2 (33.3) | 0 | 1 (16.7) | 0 | 0 | 0 | 2 (50.0) | 3 (18.8) | 0 | 9 (14.5) |
| Fall | 1 (16.7) | 1 (16.7) | 0 | 1 (16.7) | 0 | 1 (33.3) | 2 (20.0) | 0 | 3 (18.8) | 0 | 9 (14.5) |
| Urinary tract infection | 0 | 1 (16.7) | 1 (20.0) | 1 (16.7) | 0 | 1 (33.3) | 1 (10.0) | 0 | 3 (18.8) | 1 (50.0) | 9 (14.5) |
| Alanine aminotransferase  increased | 3 (50.0) | 0 | 2 (40.0) | 1 (16.7) | 0 | 1 (33.3) | 1 (10.0) | 0 | 0 | 0 | 8 (12.9) |
| Dyspepsia | 3 (50.0) | 1 (16.7) | 1 (20.0) | 1 (16.7) | 0 | 0 | 0 | 0 | 2 (12.5) | 0 | 8 (12.9) |
| Headache | 3 (50.0) | 1 (16.7) | 1 (20.0) | 2 (33.3) | 0 | 0 | 0 | 0 | 1 (6.3) | 0 | 8 (12.9) |
| Muscle spasms | 3 (50.0) | 0 | 0 | 1 (16.7) | 0 | 1 (33.3) | 1 (10.0) | 1 (25.0) | 1 (6.3) | 0 | 8 (12.9) |
| Insomnia | 1 (16.7) | 1 (16.7) | 0 | 1 (16.7) | 0 | 0 | 3 (30.0) | 1 (25.0) | 0 | 0 | 7 (11.3) |
| Pneumonia | 1 (16.7) | 0 | 0 | 0 | 0 | 1 (33.3) | 1 (10.0) | 0 | 4 (25.0) | 0 | 7 (11.3) |
| Rash | 1 (16.7) | 0 | 1 (20.0) | 0 | 0 | 1 (33.3) | 2 (20.0) | 0 | 2 (12.5) | 0 | 7 (11.3) |

Includes AEs with an onset date on or after the date of the first treatment dose and up to and including 30 days following the date of the last dose. AE, adverse event; bid, twice daily; MedDRA, Medical Dictionary for Regulatory Activities; qd, once daily

**Supplementary Table S2** Most common (≥10% of patients) treatment-related adverse events

| **AEs, n (%)** | **Treatment groups** | | | | | | | | | | |
| --- | --- | --- | --- | --- | --- | --- | --- | --- | --- | --- | --- |
|  | **bid 1 (n=6)** | **bid 2 (n=6)** | **qd 1.1 (n=5)** | **qd 1.2 (n=6)** | **qd 2.1  (n=4)** | **qd 2.2  (n=3)** | **qd 2.3  (n=10)** | **qd 3.1  (n=4)** | **qd 3.2  (n=16)** | **qd 3.3  (n=2)** | **Total  (N=62)** |
| Any treatment-related AE | 6 (100) | 6 (100) | 5 (100) | 6 (100) | 2 (50.0) | 3 (100) | 8 (80.0) | 2 (50.0) | 16 (100.0) | 2 (100.0) | 56 (90.3) |
| Diarrhea | 2 (33.3) | 5 (83.3) | 1 (20.0) | 2 (33.3) | 1 (25.0) | 2 (66.7) | 7 (70.0) | 0 | 9 (56.3) | 2 (100.0) | 31 (50.0) |
| Nausea | 3 (50.0) | 3 (50.0) | 1 (20.0) | 4 (66.7) | 0 | 2 (66.7) | 2 (20.0) | 2 (50.0) | 7 (43.8) | 2 (100) | 26 (41.9) |
| Fatigue | 5 (83.3) | 4 (66.7) | 1 (20.0) | 1 (16.7) | 0 | 2 (66.7) | 2 (20.0) | 1 (25.0) | 8 (50.0) | 0 | 24 (38.7) |
| Vomiting | 1 (16.7) | 2 (33.3) | 0 | 3 (50.0) | 0 | 2 (66.7) | 1 (10.0) | 0 | 4 (25.0) | 1 (50.0) | 14 (22.6) |
| Anemia | 0 | 1 (16.7) | 0 | 2 (33.3) | 0 | 1 (33.3) | 1 (10.0) | 0 | 6 (37.5) | 0 | 11 (17.7) |
| Decreased appetite | 2 (33.3) | 2 (33.3) | 0 | 1 (16.7) | 0 | 0 | 2 (20.0) | 0 | 4 (25.0) | 0 | 11 (17.7) |
| Dehydration | 2 (33.3) | 4 (66.7) | 0 | 1 (16.7) | 0 | 1 (33.3) | 2 (20.0) | 0 | 1 (6.3) | 0 | 11 (17.7) |
| Thrombocytopenia | 1 (16.7) | 0 | 0 | 0 | 0 | 1 (33.3) | 3 (30.0) | 1 (25.0) | 3 (18.8) | 1 (50.0) | 10 (16.1) |
| Myalgia | 1 (16.7) | 0 | 1 (20.0) | 3 (50.0) | 0 | 0 | 2 (20.0) | 1 (25.0) | 1 (6.3) | 0 | 9 (14.5) |
| Neutropenia | 0 | 2 (33.3) | 1 (20.0) | 0 | 0 | 0 | 1 (10.0) | 0 | 4 (25.0) | 1 (50.0) | 9 (14.5) |
| Abdominal pain | 1 (16.7) | 0 | 0 | 1 (16.7) | 1 (25.0) | 0 | 0 | 1 (25.0) | 2 (12.5) | 1 (50.0) | 7 (11.3) |
| Asthenia | 2 (33.3) | 0 | 2 (40.0) | 0 | 0 | 1 (33.3) | 0 | 0 | 2 (12.5) | 0 | 7 (11.3) |
| Constipation | 1 (16.7) | 1 (16.7) | 1 (20.0) | 1 (16.7) | 0 | 1 (33.3) | 2 (20.0) | 0 | 0 | 0 | 7 (11.3) |
| Dyspepsia | 3 (50.0) | 1 (16.7) | 0 | 1 (16.7) | 0 | 0 | 0 | 0 | 2 (12.5) | 0 | 7 (11.3) |

Includes AEs with an onset date on or after the date of the first adavosertib dose and up to 30 days, inclusive, following the date of the last dose of adavosertib

**Supplementary Figure S1** Biomarker analysis for the genomic patient profiles

**
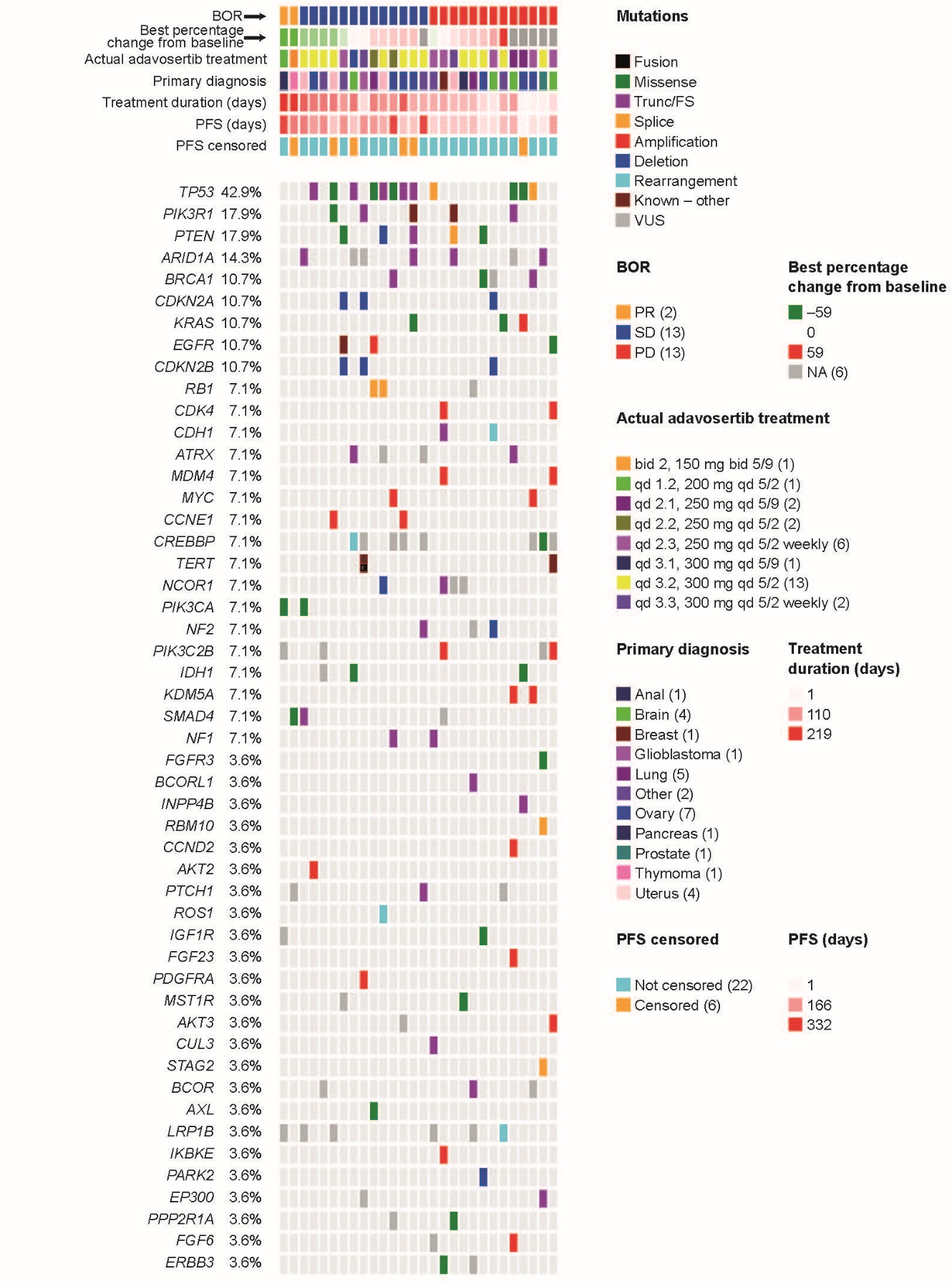
**

Tumor tissue was analysed using the Foundation Medicine (FMI) NGS platform. The analyses included all patients who consented to the sample collection and for whom a valid test result was obtained (n=28).

Supplementary Figure S2 Geometric mean (± SD) plasma concentrations of adavosertib versus time at the RP2D


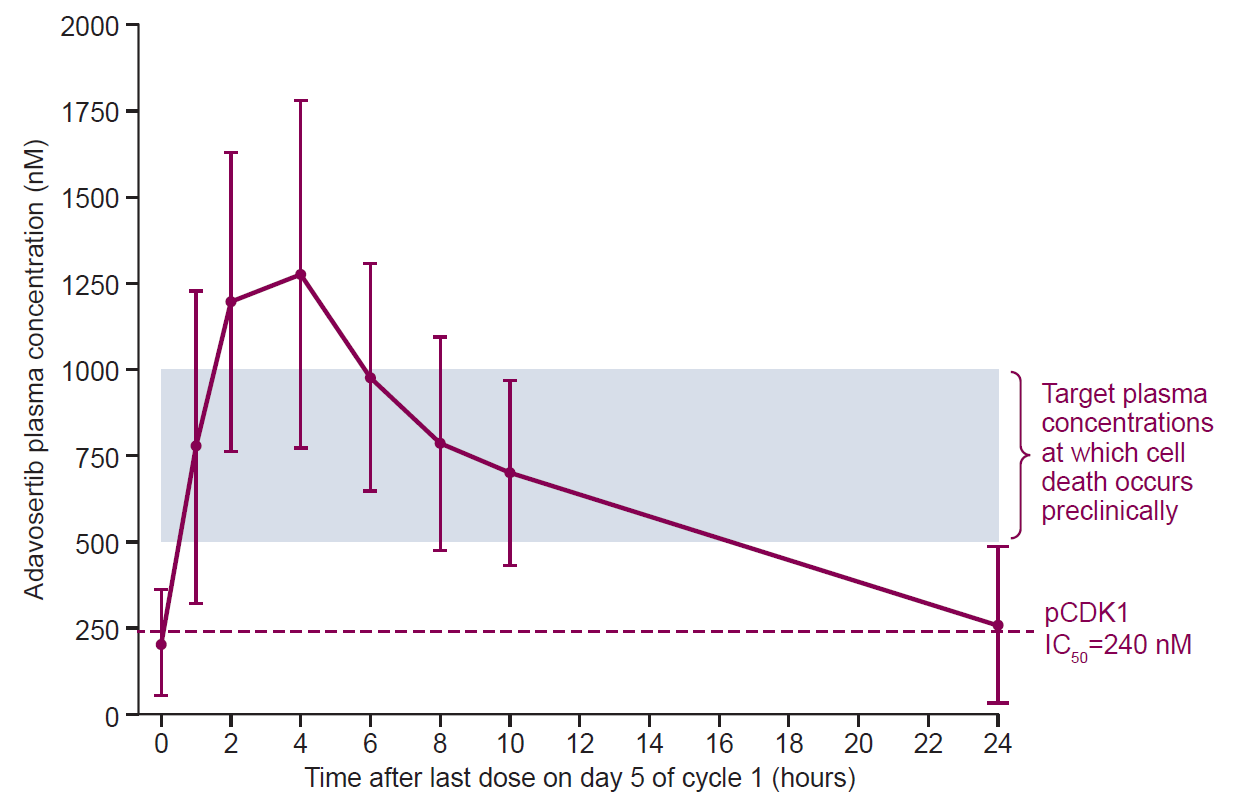


Data from all three 300 mg cohorts (qd 3.1–3; n=21) are included; data are shown as geometric mean ± standard deviation (SD)

Supplementary Figure S3 Dose proportionality (cycle 1, day 1) for (a) AUC_0–10_ and
(b) C_max_


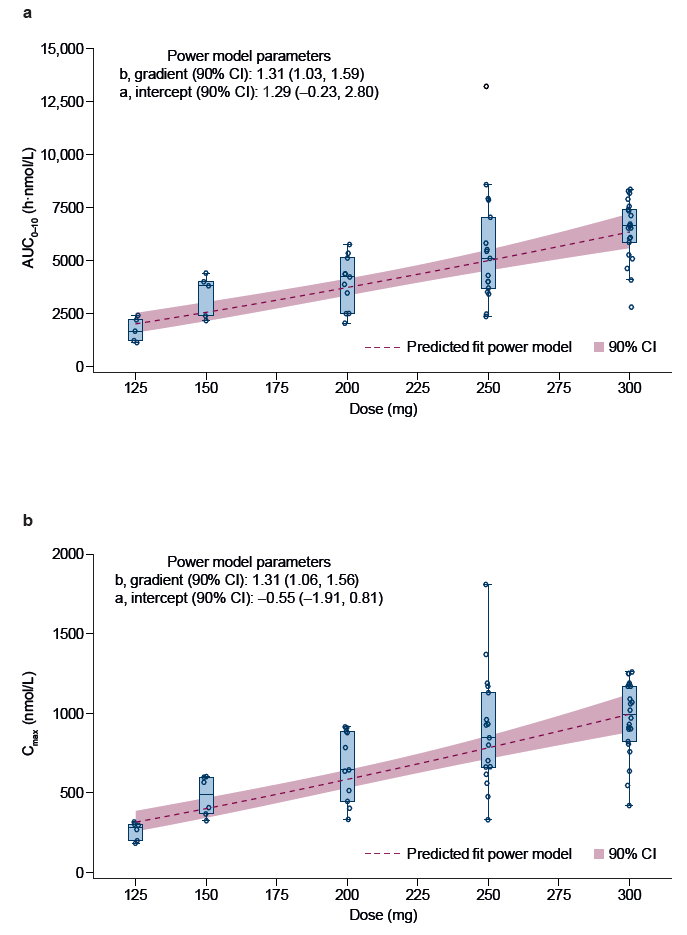

Supplement: Supplementary file 4 — Supplementary Material 4 [file 10637_2023_1371_MOESM4_ESM.docx]
